# Plastrum Testudinis Extract Mitigates Thiram Toxicity in Broilers via Regulating PI3K/AKT Signaling

**DOI:** 10.3390/biom9120784

**Published:** 2019-11-26

**Authors:** Hammad Qamar, Muhammad Waqas, Aoyun Li, Mudassar Iqbal, Khalid Mehmood, Jiakui Li

**Affiliations:** 1 College of Veterinary Medicine, Huazhong Agricultural University, Wuhan 430070, China; hammadqamaran@gmail.com (H.Q.); muhammadwaqas@upr.edu.pk (M.W.); mudassar.iqbal@webmail.hzau.edu.cn (M.I.); khalid.mehmood@webmail.hzau.edu.cn (K.M.); 2 Faculty of Veterinary & Animal Sciences, University of the Poonch, Rawalakot, District Poonch 12350, Azad Jammu & Kashmir, Pakistan; 3 University College of Veterinary & Animal Sciences, The Islamia University of Bahawalpur, Bahawalpur 63100, Pakistan; 4 College of Animals Husbandry and Veterinary Medicine, Tibet Agricultural and Animal Husbandry University, Linzhi, Tibet 860000, China

**Keywords:** broiler, toxicity, Chinese traditional medicine, liver oxidative stress, gene expression, bone metabolic disorder

## Abstract

Tibial dyschondroplasia (TD) negatively affects broilers all over the world, in which the accretion of the growth plate (GP) develops into tibial proximal metaphysis. Plastrum testudinis extract (PTE) is renowned as a powerful antioxidant, anti-inflammatory, and bone healing agent. The current study was conducted to evaluate the efficacy of PTE for the treatment of thiram-induced TD chickens. Broilers (day old; *n* = 300) were raised for 3 days with normal feed. On the 4th day, three groups (*n* = 100 each) were sorted, namely, the control (normal diet), TD, and PTE groups (normal diet+ thiram 50 mg/kg). On the 7th day, thiram was stopped in the TD and PTE group, and the PTE group received a normal diet and PTE (30 mg/kg/day). Plastrum testudinis extract significantly restored (*p* < 0.05) the liver antioxidant enzymes, inflammatory cytokines, serum biochemicals, GP width, and tibia weight as compared to the TD group. The PTE administration significantly increased (*p* < 0.05) growth performance, vascularization, *AKT* (serine/threonine-protein kinase), and *PI3K* expressions and the number of hepatocytes and chondrocytes with intact nuclei were enhanced. In conclusion, PTE has the potential to heal TD lesions and act as an antioxidant and anti-inflammatory drug in chickens exposed to thiram via the upregulation of *AKT* and *PI3K* expressions.

## 1. Introduction

Pesticides are a group of chemicals being utilized in agriculture for the eradication of weeds, pests, and diseases. However, because of their integration into living things directly or indirectly via air, soil, water or the food chain, their extensive usage is associated with toxicity in humans, livestock, and poultry [1]. Although pesticides are frequently poisonous to other organisms, their detrimental effects depend on the type of species [2]. Thiram, a dithiocarbamate, is commonly utilized as a pesticide or fungicide in agricultural farming, especially for treating grains aimed for the seed protection during preservation or used as storage for food consumption [3]. However, thiram as a plant pesticide has been banned for foliar application in the EU since March 2019 and will be banned for other applications starting from January 2020. If a thiram-containing diet is offered to avian species, it can have a remarkable and damaging effect and can lead to tibial bone metabolic disorder known as tibial dyschondroplasia (TD) [4]. Broiler chickens have a significantly high prevalence of TD due to the fact of thiram-contaminated feed [5], since there is more application of thiram in the food and agriculture sector; thus, due to a contaminated environment, it may become part of the feed [6]. Poultry birds and other model animals show the same symptoms of TD as in naturally occurring cases of TD [7,8]. Thiram toxicity results in immune disorder, bone growth reduction and cartilage cells destruction by adhering with cell membrane due to its lipophilic nature. In severe cases, it can lead to angiogenesis inhibition, permanent membrane damage, and inactivation of bone morphogenic proteins [6]. Tibial dyschondroplasia (TD) is a common bone problem in broilers characterized by non-vascularization and non-mineralization in tibia growth plates [9] which generally leads to lameness and uncharacteristic growth of chondrocytes [10,11,12]. Normally, division of cells takes place in the proliferative zone which is responsible for the initial development of longitudinal bone, and chondrocytes are involved in bone remodeling, cartilage vascularization, and ossification [13]. However, TD lesion is a manifestation of endochondral ossification halt, proliferation of cartilage cells, and development of avascular cartilage mass in the tibia metaphysis region [4,14,15]. As the mechanism of TD is not fully understood yet, new insights are necessary for control and treatment of this disease.

Tumor necrosis factor (TNF) is a type of small protein that has a pleiotropic pro-inflammatory effect and can significantly trigger the process of bone resorption and inhibition of bone development [16]. It possesses two receptors, TNF receptor 1 and 2; however, the latter one reveals more affinity to TNF [17]. After the activation of TNFR2 by membrane-bound TNF, TNF receptor-associated factor 2 (TRAF2) is recruited which leads to the activation of the phosphatidylinositol 3-kinase (PI3K)/AKT signaling pathway [18].

Current studies regarding PI3K/AKT signaling have exposed that it critically regulates the process of endochondral ossification [19,20]. During osteogenesis the AKT plays a pivotal role in maintaining forkhead proteins (FOXOs) which affect bone development and survival of osteoblasts [21,22]. Moreover, AKT is also involved in the differentiation of osteoblasts from mesenchymal stromal cells (MSCs) in collaboration with bone morphogenetic protein 2 (BMP2) [23].

Plastrum testudinis (PT) is a vital traditional Chinese drug possessing strong bone healing ability, a common clinical practice for eradicating bone diseases in China. Turtles possess an incredible skeleton structure; the dorsal peripheral is the combination of about 50 bones and is known as a carapace, whereas the ventral surface is called the plastron fusion of nine bones. Plastrum testudinis is organic in nature and present in the plastron and carapace of a turtle found in China (*Chinemys reevesii*; Fam. Emydidae). It is believed that the shell’s cells’ developmental process might be guided by bone morphogenetic proteins (BMPs) and signaling proteins such as (fibroblast growth factor) FGF [24]. A plastrum testudinis extract (PTE) contained about nine different compounds including two carboxylic acids (stearic acid and palmitic acid), three steroids (cholesterol, 4-cholesten-3-one and cholesterol myristate), and four esters (methyl palmitate, ethyl palmitate, methyl stearate, and ethyl stearate) [25]. These vital compounds are responsible for the regulation of cell proliferation as they belong to important diversified pharmaceutical classes [25]. Steroids at low levels are involved in the proliferation of bone precursors (i.e. osteoblasts) [26] whereas fatty acids promote alkaline phosphatase (ALP) activity, cells growth, survival, and proliferation by regulating proliferative related genes [27,28,29]. Palmitic acid and its derivatives promote IgA responses by regulating inflammatory cytokines [30] and initiate cells proliferation via p38 MAPK/ERK-Akt signaling [31]. Stearic acid provides protection against hepatotoxicity by regulating serum biochemicals level [32], reduces inflammation by suppressing NF-κB activity, and enhances antioxidant enzymes in cerebral neurons via the up-regulation of PPARγ [33,34,35]. Cholesterol and its derived steroids play a crucial role in bone metabolism and homeostasis, and their endogenous physiological levels are indispensable for bone MSC osteogenesis [36]. Methyl stearate and methyl palmitate possesses antioxidant properties and provide protection against DPPH-induced oxidation by scavenging free radicals [37]. Plastrum testudinis extract can endorse the multiplication and growth of bone cells and possess significant antioxidant property [38,39]. 

However, its use in the poultry industry for disease control with special reference to tibial dyschondroplasia has not yet been studied. Keeping in mind the beneficial effects of PTE, a scientific trial was conducted to evaluate the potential use of PTE against thiram toxicity in chickens via the AKT/ PI3K signaling pathway, and it provides new insights for better understanding of the molecular pathology and treatment of disease.

## 2. Materials and Methods

### 2.1. Reagents and Compounds

Thiram was obtained from Shanghai Biochemical Company, Shanghai, China. Plastrum testudinis extract (PTE) was bought from the Shandong Jin Sheng Biotechnology Co. Ltd., Shandong, China. All further viable kits and reagents were procured from Jiancheng Co. Ltd., Nanjing, China.

### 2.2. Approval of Ethics

The experimental plan was approved from the Research and Animal Welfare wing, Ethics Committee of Huazhong Agricultural University, Wuhan, China and guidelines were strictly followed (approval no. 31273519).

### 2.3. Experimental Plan

Commercial broiler strain Arbor Acres (AA) (day-old chicks; *n* = 300) were procured from a local hatchery (Chia Tai Animal Company, Jingzhou, China). The sex of all the birds used in this study was male. The chickens were housed in different metal cages with provision of good care, hygienic environment and the standard temperature, and raised for 18 days. To avoid any distress and injury, the chicks were raised with care and strictly observed during the entire experiment. Moreover, until the end of the experiment, not a single bird showed any type of breathing discomfort or illness. A standard diet as directed by National Research Council [40] was available to the chicks around the clock. Three groups, the control, tibial dyschondroplasia (TD), and plastrum testudinis extract (PTE), were made containing an equal number of animals, i.e., (*n* = 100). The control group received a basal diet from day one until the end of the experiment whereas thiram was added in the basal diet of the TD and PTE groups at 50 mg/kg [41] during a 3–7 day interval, and, on the rest of the days, they received a normal diet. The PTE was supplied daily to the chickens orally from day 8–18 in the PTE group at 30mg/kg [38,42].

### 2.4. Analyses of Production Parameters and Tibia Parameters

Production parameters (i.e., total weight of chicks, feed conversion ratio, and daily feed consumed) and mortality were recorded during the research trial. At four different time points (day 7, 10, 14, and 18), humane slaughtering of chickens (*n* = 20 from each group) was done (pentobarbital at 25 mg/kg was injected before euthanization) [43], and then the tibia bone growth plate (GP) size (measured using digital caliper), TD score (from 0 to 4 according to lesion severeness as per our previous experiment) [44], and tibia parameters (i.e., length, width, and weight were noticed), then, a few tibial bones were deposited at −80 °C for further analyses [44].

### 2.5. Serum Biochemicals Analysis

Before slaughtering, the cardiac puncture was done [9] for the collection of blood samples (*n* = 20) from each group at four different days as mentioned above, and, then, they were subjected to centrifugation at 3000× *g* for 20 min to obtain blood serum and stored at −70 °C. The analysis of ALP, aspartate aminotransferase (AST), and alanine aminotransferase (ALT) levels (calculated in U/L; unit per litre) from an individual treatment was conducted along with the determination of TNF-α and IL-6 (measured in picogram per milliliter, pg/mL) using commercial kits following the manufacturer’s protocol [45,46]. The ALP/AST/ALT levels were assessed using a biochemical analyzer, while cytokines were analyzed with the ELISA method.

### 2.6. Liver Weight and Antioxidants Analysis

Calculation of glutathione peroxides (GSH-Px), total antioxidant capacity (T-AOC), superoxide dismutase (SOD), and malondialdehyde (MDA) levels was done from liver samples (*n* = 20) collected and weighed at four different time points, as mentioned before, from an individual group. The first three parameters were calculated in U/mg (unit per milligram) of protein, whereas the latter one was calculated in nmoles/g (nanomoles/gram). The liver samples’ collection and preparation was done according to procedure described in previous experiment and liver antioxidants were assessed with UV spectrophotometer using commercial kits [45].

### 2.7. Hematoxylin and Eosin (H&E) Staining and Immunohistochemistry

The individual tibiotarsal bones (*n* = 20) from each group on day 7, 10, 14, and 18 were preserved simultaneously in phosphate buffered saline (PBS) and 4% paraformaldehyde at 4 °C on various days as mentioned above. Decalcification was done with 10% ethylenediamine tetra acetic acid, then dehydrated in ethanol, washed out in xylene, and covered with paraffin wax. A 4 μm thick incision was made to prepare the slides. These paraffin-embedded segments were dewaxed in xylene and stained with H&E stain [47]. A light microscope was used to observe the pathological changes in the tibial bones. For histopathological observation of liver on day 18, 10% neutral buffered formalin was used to fix the liver tissues, then embedded and washed with paraffin and xylene, then cut into 5 µm small parts and used for H&E staining [48].

For immunohistochemistry analysis (*n* = 20 individual tibiotarsal bones per group selected randomly on day 18 only), the PBS and peroxidase blocking solution (Boster, Wuhan) were used for washing the slides, and then primary antibody treatment for AKT (1:100; Affinity AF6261) and PI3K (1:300; BIOSS BSM-33219m) was done at 4 °C overnight. Secondary antibody treatment was done using horseradish peroxidase-conjugated anti-rabbit antibody in the dark for 2 h at 25 °C. Diaminobenzidine (DAB) was used for chromogen staining. The light microscope was used to observe the changes and Image-Pro ® Plus 6.0 software (Media Cybernetics, Rockville, MD, USA) was used to analyze the images for the calculation of vascular area, bone trabecular ratio and number of cells [6,44,49].

### 2.8. RNA Extraction and RT-qPCR

The RNA extraction from tibial GP (*n* = 20) from each group was done using the Trizol extraction method (Invitrogen, Carlsbad, California, USA). The integrity of RNA was analyzed via denaturing formaldehyde gel electrophoresis, and Nanodrop 2000 analyzer (Thermo Fisher Scientific) was used to check the purity and concentration. The cDNA was synthesized using a commercial kit (first-strand reverse transcription cDNA kit, Tian Gen, China) according to the manufacturer’s specifications. A 20 μL, the reaction mixture containing oligo (dT)18, 2× reaction Mix, and 5 μL RNA was used for reverse transcription in a thermocycler (Applied Biosystem CA, USA) at the reaction temperature of 42 °C for 30 min and 85 °C for 5 min [50]. Primer Premier Sofware version 5.0 (Wuhan biotechnology Co. Ltd., Wuhan, China) was used to design the gene specific primers from already reported sequences for respective genes [39] (Table 1). Quantitative RT-PCR was performed using Step One-Plus TM Real-Time PCR System (Applied Biosystems, Foster City, CA, USA) in quadruplex. Reaction conditions used for RT-PCR were the same as reported in our previous experiment [49]. Reference gene *GAPDH* was used to determine relative expression of target genes through RT-PCR using∆∆CT method [47].

### 2.9. Western Blot Analysis

The tibial bone GPs (*n* = 9) were used for the Western blot (WB) analysis. The WB analysis was done according to the method explained by Mehmood et al. [44]. In short, the GP samples’ homogenization was done in PBS and subjected to a 4 °C cold environment for 2 h. Then centrifugation was done at 14,000 rpm for 10 min and SDS-PAGE was performed on 10% polyacrylamide gel to obtain the equivalent quantity of proteins. The incubation of membranes was done for 1.5 h in 5% skimmed milk and subjected to the primary antibody (rabbit polyclonal anti-AKT, 1:1000; Affinity AF6261 and mouse monoclonal anti-PI3K, 1:1000; BIOSS BSM-33219m) at 4 °C for overnight. Saline with 0.1% Tween 20 was used for 5 min to wash the membranes, and then they were subjected to the secondary antibody for 30 min (goat anti-mouse, 1:3000) at normal temperature. Once again, the washing of membranes was done with saline and images were taken using a standard imaging device luminescent Image Analyzer (CA, USA).

### 2.10. Statistical Analysis

The normal distribution of data and equality of variances was assessed using the Shapiro–Wilk test and Levene’s test, respectively [51,52]. The ANOVA test was performed on SPSS19.0 for the analyses of data and comparison was done using the Student’s *t*-test. All data are expressed as the mean ± SD. If *p* < 0.05, then the results were regarded as significant.

## 3. Results

### 3.1. Production Parameters

**Figure 1:**
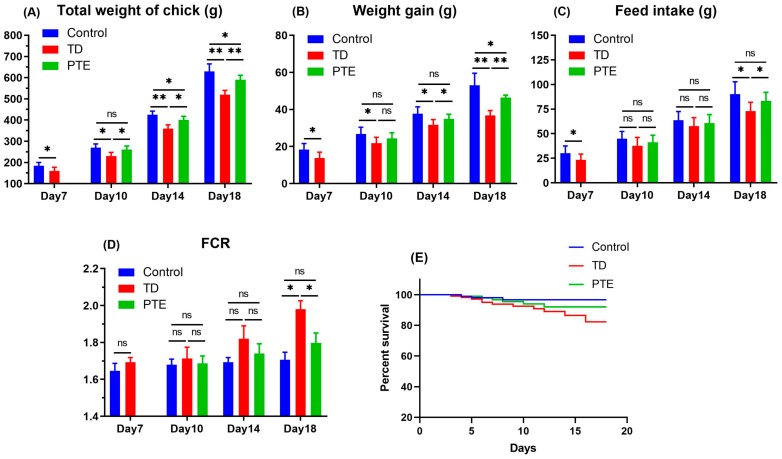
Growth performance parameters. (**A**) The average weight of the chicken; (**B**) average weight gain; (**C**) daily feed consumption; (**D**) feed conversion ratio (FCR), and (**E**) percent survival among three groups. TD: Tibial dyschondroplasia. PTE: Plastrum testudinis extract. The data are shown as the mean ± SD. ** *p* < 0.01. * *p* < 0.05.

The growth performance results revealed that the TD and PTE groups had a positive correlation between daily feed consumption and the weight of the chickens, while there was a negative association in FCR. The PTE group had significantly better (*p* < 0.05) FCR, total body weight, weight gain, and feed consumption as compared to the TD chickens on day 18. The FCR was significantly poor (*p* < 0.05) in the chickens exposed to thiram as compared to the control and PTE groups on day 18 which indicates lower weight gain and feed consumption. Although, administration of PTE was significantly reduced (*p* < 0.05), the FCR was near to the control group’s level on day 18. No significant differences were found in death rate and survival percentage among the three groups (Figure 1).

### 3.2. Effect of PTE on Growth Plate Size, Tibia Parameters, and TD Score

**Figure 2:**
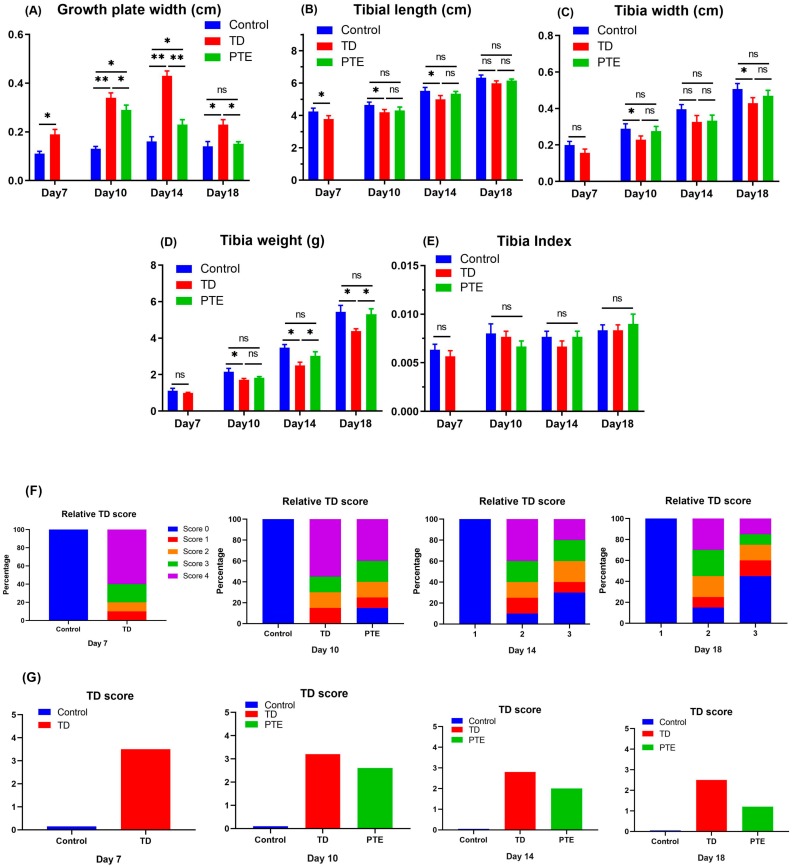
Tibia parameters and TD score. (**A**) Growth plate size of tibial bone at different days; (**B**) length of tibial bone; (**C**) width of tibial bone; (**D**) weight of tibial bone; (**E**) tibia index; (**F**) relative TD score; and (**G**) average TD score. The data are presented as the mean ± SD. ** *p* < 0.01. * *p* < 0.05.

Thiram had a negative effect on bone GP size and it eventually led to the enhancement of the GP size (Figure 2). On day 10 and 14, the GP size in TD chicks was significantly high (*p* < 0.05) as compared to other groups. After the provision of PTE, the GP size decreased gradually as compared to TD chicks, and, on day 18, it was comparable to the control group. The TD score also decreased as PTE was administrated in TD chicks on day 14 and 18. Chickens affected by TD disease exhibited significantly reduced (*p* < 0.05) tibia weight in comparison with chickens in the control group, but administration of PTE reinstated the tibia weight.

### 3.3. Biochemical Criterion Analysis of Serum and Liver Histopathology

**Figure 3:**
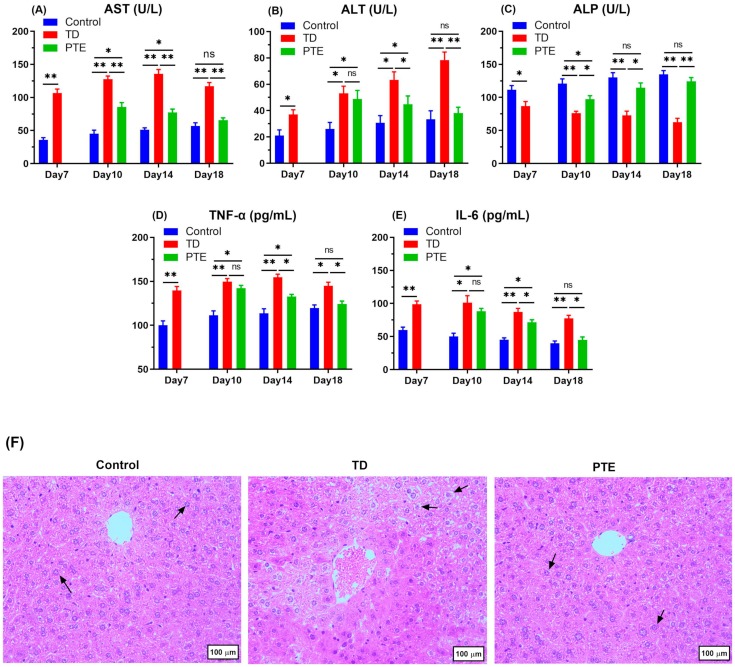
Serum biochemical analysis of chickens at a different age. (**A**) Aspartate aminotransferase (AST) analysis; (**B**) alanine aminotransferase (ALT) analysis; (**C**) alkaline phosphatase (ALP) analysis; (**D**) tumor necrosis factor analysis (TNF-α); and (**E**) interleukin 6 (IL-6) analysis. (**F**) H&E was done to observe the histopathological changes in the livers of the control, TD, and PTE groups. Arrows indicates normal hepatocytes in the control and PTE groups, whereas karyopyknosis can be seen in the TD group. The data of all groups are expressed as the mean ± SD. ** *p* < 0.01.* *p* < 0.05.

Serum analysis revealed that ALP content decreased (*p* < 0.05), and AST and ALT levels increased continuously after the onset of TD in TD chickens as compared to healthy chicks (Figure 3). However, provision of PTE to TD chickens reverted the ALP, ALT, and AST measurements. The ALT and AST levels declined significantly (*p* < 0.05) after the PTE administration on day 10 and 14 in comparison with the TD group; however, non-significant results were obtained on day 18 when comparison was done with the control group. The PTE significantly increased (*p* < 0.05) the ALP level on day 10, and the measurement was almost the same as the control chickens on day 14 and 18. Both IL-6 and TNF-α were enhanced significantly (*p* < 0.05) in broilers exposed to thiram in comparison with broilers in the control group, whereas PTE provision significantly lowered (*p* < 0.05) the blood serum TNF-α and IL-6 in comparison with TD chickens on day 14 and 18. These results indicate the disturbance in the liver’s normal metabolic activities and liver damage. The control group had the normal arrangement of cells, whereas in the TD group, asymmetrical arrangement of hepatocytes and karyopyknosis was seen. After PTE administration, the irregular cells’ arrangement vanished and hemorrhages in hepatocytes were reduced (Figure 3F).

### 3.4. Liver Weight and Determination of Antioxidant Status in Liver

**Figure 4:**
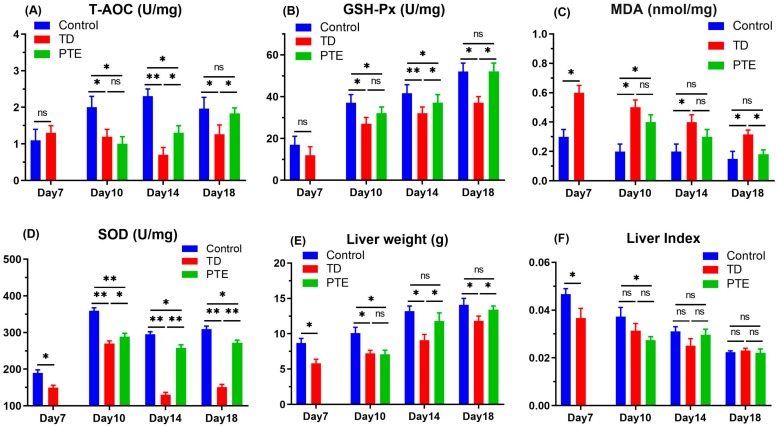
Liver weight and antioxidant enzymes analysis. The level of (**A**) total antioxidant capacity (T-AOC); (**B**) glutathione peroxidase (GSH-Px); (**C**) malondialdehyde (MDA); (**D**) superoxide dismutase (SOD). (**E**) Liver weight and (**F**) liver index were checked and are shown as the mean ± SD. * *p* < 0.05; ** *p* < 0.01.

For the assessment of hepatic toxicity, the amount of T-AOC, SOD, GSH-Px, and MDA were measured. The levels of the first three antioxidants declined significantly (*p* < 0.05) in TD chickens when compared with the control group, while MDA increased significantly (*p* < 0.05). However, PTE administration significantly enhanced (*p* < 0.05) SOD, T-AOC, and GSH-Px enzymes whereas it remarkably reduced (*p* < 0.05) MDA enzymes (Figure 4). Tibia dyschondroplasia (TD) highly affected the chickens’ livers weight. On all days, the liver weight of TD affected birds was significantly reduced (*p* < 0.05) in comparison with the control group. After the administration of PTE, liver weight was significantly improved (*p* < 0.05) on day 14 and 18 in comparison with chickens in the TD treatment. The liver index among the PTE and TD groups was non-significant (Figure 4).

### 3.5. Histopathological Investigation of the Growth Plates of the Tibia

**Figure 5:**
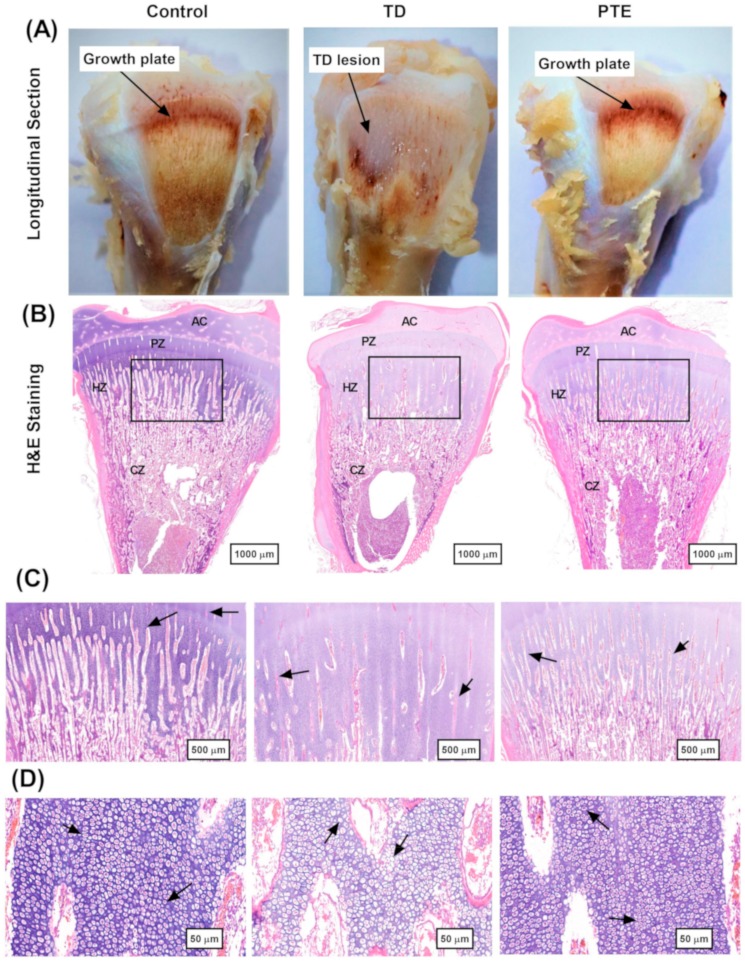

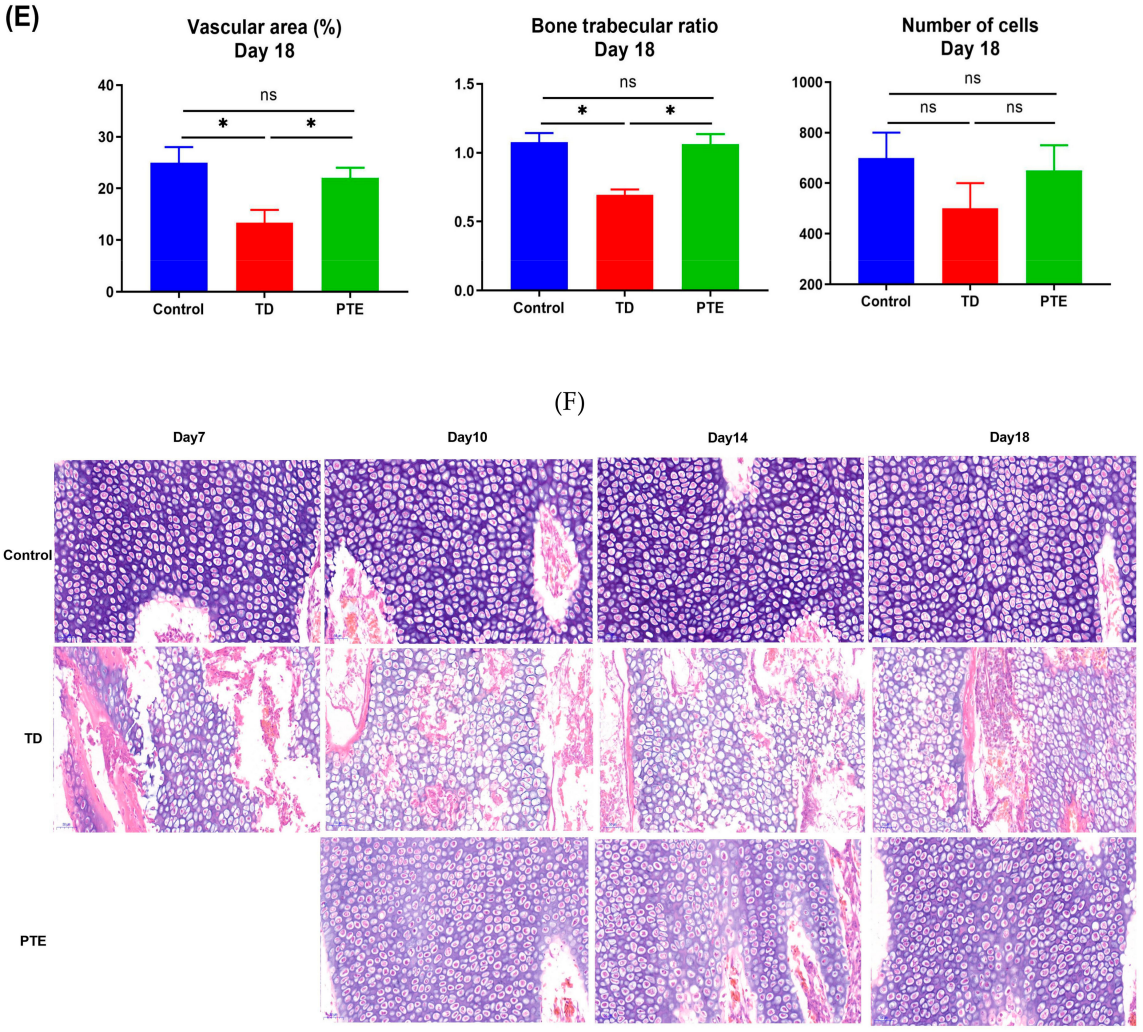
Histopathological investigation of the growth plates of the tibia. (**A**) Longitudinal section of control, TD, and PTE bones on day 18. (**B**) H&E staining of bones at day 18 depicts different regions, i.e., AC (articular cartilage), PZ (proliferative zone), HZ (hypertrophic zone), and CZ (calcified zone). The rectangular area shows less vascularity in the TD group. (**C**) Black arrows show normal trabecular bone in the control group and abnormal trabecular bone in the TD group. (**D**) Chondrocytes having a disintegrated and dissolved nucleus can be seen in the TD group, but the control and PTE groups had well-arranged columns of chondrocytes with intact nuclei. (**E**) Images were analyzed using Image-Pro^®^ Plus 6.0 software for the calculation of vascular area, bone trabecular ratio, and number of cells on day 18. (**F**) The control group had a normal arrangement of cells whereas, in the TD group, dead cells and an abnormal arrangement of cells can be seen. However, after PTE supplementation, the number of chondrocytes with intact nuclei in PTE bones was similar to that of the control bones and much greater than TD bones.

Histological investigation showed that the control group exhibited a well-established network of blood vessels and well-arranged columns with intact cells. On the other hand, TD chicks exhibited distorted and necrotized cells and abnormal arrangement of chondrocytes with reduced vascular area. The PTE administration alleviated the adverse effects of thiram and increased in angiogenesis; the vascular area was observed in the hypertrophic region which restored the chondrocytes’ arrangement, and the number of chondrocytes with intact nuclei in PTE bones was similar to that of the control bones and much greater than TD bones. The length of trabecular bone was reduced significantly (*p* < 0.05) in the TD group as compared to the chickens in the control group. After PTE administration to the chickens, trabecular bone length was restored and improved remarkably (*p* < 0.05) as compared to TD affected birds (Figure 5).

### 3.6. Immunohistochemistry of Tibial Growth Plates

**Figure 6:**
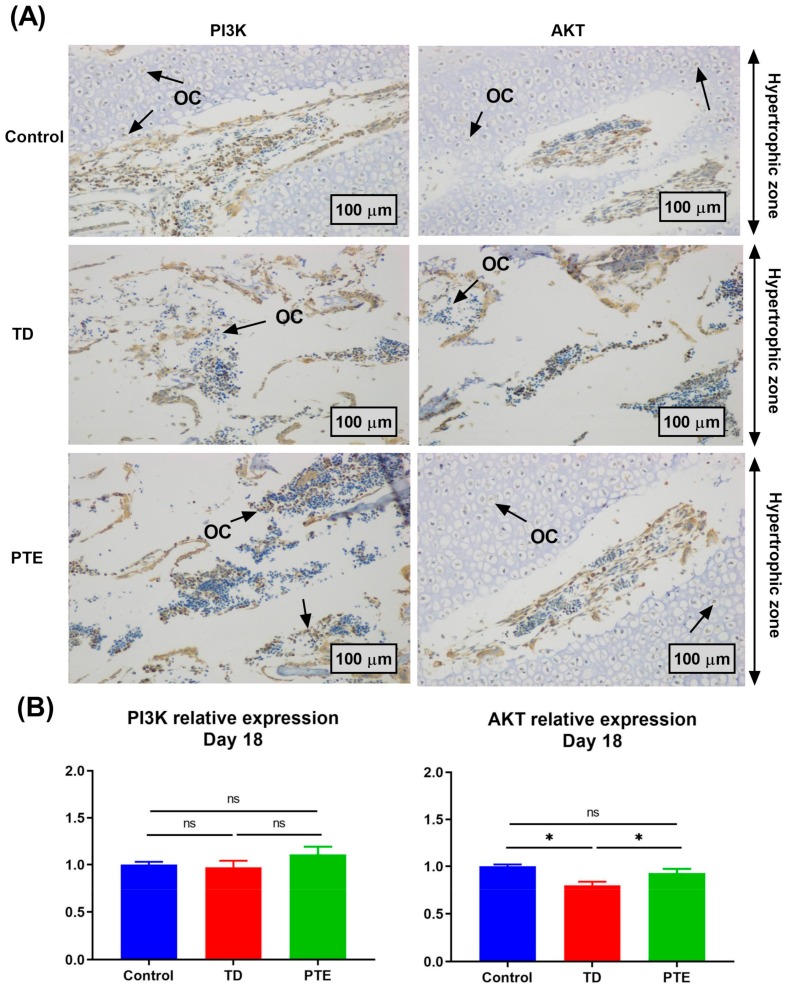
Immunohistochemistry of AKT and PI3K in the growth plate. (**A**) Immunohistochemical expression of PI3K and AKT relative to the control. The TD chicks showed lower expression of AKT antibody-stained cells whereas, after PTE administration, the AKT antibody-stained cells increased on day 18 as indicated by the arrows. The arrows indicate osteocytes (OCs) within the hypertrophic zone. (**B**) The immunohistochemistry images were analyzed using Image-Pro^®^ Plus 6.0 software.

The immunohistochemistry was done to check the antibody expression of AKT and PI3K in tibia GP. The thiram group had fewer cells positively stained with AKT antibody as compared to healthy chickens, while the PTE administration reversed the results and AKT antibody stained cells were increased as compared to the TD affected chicks. No significant differences were found in PI3K localization among the control, TD, and PTE groups (Figure 6).

### 3.7. AKT and PI3K Genes and Protein Expression in the Growth Plate

**Figure 7:**
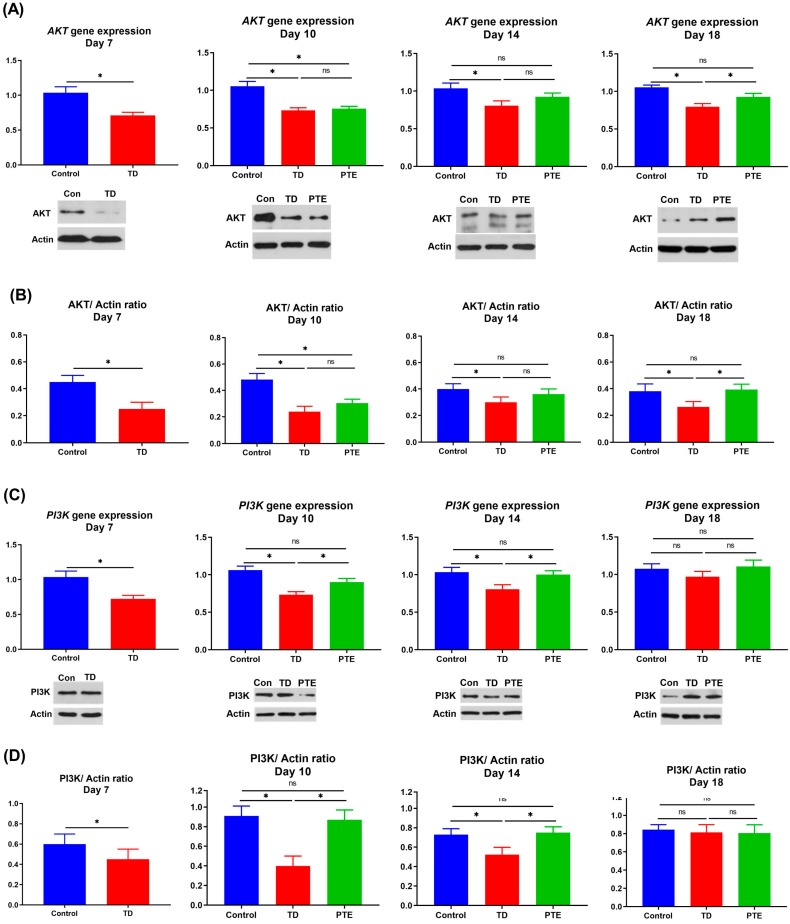
Fold change in the *AKT* and *PI3K* genes and protein expression. (**A**) The *AKT* gene and protein expression on various days. (**B**) Images were analyzed using Image-Pro ^®^ Plus 6.0 software to evaluate the AKT/Actin ratio. (**C**) The *PI3K* gene and protein expression on various days. (**D**) Images were analyzed using Image-Pro^®^ Plus 6.0 software to evaluate the PI3K/Actin ratio. The *AKT* and *PI3K* expression decreased in the TD group whereas PTE administration increased the gene and protein expressions. The data are expressed as the mean ± SD. * *p* < 0.05.

The *AKT* and *PI3K* genes’ expression were checked through RT-qPCR. The results showed that the *AKT* mRNA was remarkably less (*p* < 0.05) expressed in TD chickens in comparison with the control group on day 7. A similar trend was seen on other days. After administration of PTE, non-significant results were obtained regarding the *AKT* gene expression on day 10 and 14 as compared to the TD group; however, the *AKT* mRNA value increased significantly (*p* < 0.05) as compared to the TD group near to the control level on day 18 (Figure 7). The *PI3K* gene expression was remarkably lower (*p* < 0.05) in TD chicks when compared with chicken in control group on day 7 (Figure 7). A similar trend was noticed on day 10 and 14. There was no difference in *PI3K* gene expression between TD chickens and PTE chickens on day 18. However, gene expression was remarkably improved (*p* < 0.05) in chicks after the administration of PTE as compared to TD chickens on day 10 and 14. Similar results were obtained for protein expression.

## 4. Discussion

Thiram is used as a pesticide in the agricultural sector; however, the toxic effects associated with thiram is of great concern to animal health. Embryonic revelation to thiram can aggravate several skeletal disorders [53]. The formation of endochondral bone is interrupted after the death of chondrocytes [54,55]. Thiram can reduce production performance and affect tibia growth in broiler chickens. It can lead to an increase in the size of growth plate measurement, and a white opaque plug is formed that hinders the minerals deposition and eventually stops the process of ossification in chickens affected from tibial dyschondroplasia [6].

Tibial dyschondroplasia is categorized into metabolic disorder and considered as the main leg problem in rapid growing broilers, affecting the proximal GP of the tibial bone. It is manifested by the increased size of the GP with non-mineralization and non-vascularization, as well as deformity in tibia bone and lameness in chicks [12,14]. Principal clinical aspects of TD reported by researchers are abnormal gait, reduced feed consumption, lameness in one or both legs, difficulty in standing, ataxia, and eventually death [4,10,56]. The poultry industry faces heavy economic losses due to the fact of TD, and it also affects the carcass quality and meat production [57,58]. In TD, there is an uncharacteristic protein excretion and apoptosis behavior exhibited by cartilage cells which lead to severe cell damage, ultimately triggering a reduction in the degradation rate of cartilage extracellular matrix, which confines the bone deposition space [59,60]. The cells halt to replicate and suffer apoptosis and hypertrophy in tibia bone. The tibial growth plate of chickens exposed to thiram exhibited a decreased number of cells and an irregular pattern in columns of the chondrocytes [61]. In TD, most of the cells located in the hypertrophic zone were smeared, metabolically arrested, had an irregular arrangement of columns, and possessed no intact nuclei [62]. Chickens affected with TD also create a problem during handling, because the lesions in birds are more prone to fractures thus they are condemned at processing plants [63]. Moreover, TD cause lameness which is also an issue regarding animal welfare [13]. The PTE is a mixture of compounds capable of curing osteoporosis and various bone disorders [38,42]; it enhances endochondral ossification by improving the bone regeneration process [39,64].

In the present study, it was evident that TD chickens exhibited poor growth performance in terms of reduced body weight and feed intake, weight gain, difficulty in movement and standing as reported in previous studies [43,45]. Conversely, after PTE administration, the chickens in the PTE group became alert and healthy, and performance indices were significantly improved (*p* < 0.05) on day 18 in comparison with the TD group. The TD chickens showed poor growth performance due to the reduced feed intake, weight gain, difficulty in movement and standing as in agreement with other literature [50]. After the administration of PTE, chickens not only become alert and healthy but also improved the feed intake, body weight, and feed conversion ratio. In sum, PTE treatment exhibited a noticeable positive influence on the overall performance of TD chickens. The PTE administration alleviated the adverse effects of thiram, and increased the angiogenesis which restored the chondrocytes’ arrangement, the network of blood vessels and bone trabecular ratio. Moreover, chondrocytes with intact nuclei were much greater in PTE bones than the TD bones as in agreement with other studies [43,45,47]. Chicks with TD exhibited scattered dead and anucleate cells, opaque cartilage, abnormal arrangement of bone cells with disintegrated and dissolved nuclei and a higher percentage of extracellular matrix as in accordance with other researchers [43,44]. This is due to the fact that avascularization in tibia, non-calcification, and increased extracellular matrix lead to a decline in angiogenesis that disturbs the nutrient transportation to developing chondrocytes [9,47]. This subsequently leads to cellular apoptosis and failure in completing the mineralization and calcification process by osteoblasts [10,65].

The serum biochemicals such as ALT, ALP, and AST are biomarkers to assess the liver metabolism, and higher AST and ALT levels indicate that there is liver damage due to the increased release of aminotransferase from liver cells into the blood [66]. The PTE decreased (*p* < 0.05) AST and ALT outflow from liver cells. Thiram exhibits a strong oxidative property and causes oxidative stress in humans by inhibiting GSH and SOD activity [67]. The MDA level increased as a result of oxidative stress in the body which damages the cell membrane via lipid peroxidation [56,68]. Our findings revealed that TD chickens exhibited liver damage because T-AOC, SOD, and GSH-Px proportions were significantly low (*p* < 0.05), while the MDA value was higher as compared to control group chickens as reported by other scientists [45,69]. However, after the administration of PTE, T-AOC, SOD, and GSH-Px, proportions increased remarkably (*p* < 0.05), while the MDA value decreased to a normal level. The PTE restored the antioxidant status of TD chickens, the it restored the antioxidant status of TD chickens, confirming that PTE is a potent antioxidant agent and can recover the antioxidant status of the animal [70].

The PI3K/AKT pathway plays a crucial role in osteogenic differentiation of bone MSCs and acts as a chief regulator in bone cells’ proliferation and metabolism [20,71,72,73]. Our study exposed for the first time that *AKT* and *PI3K* were downregulated in TD chickens and PTE upregulated the mRNA and protein expressions of AKT and PI3K. The downregulation of PI3K and AKT protein expression also has been reported in osteoporosis rats by different researchers [74,75,76]. The BMPs belong to the TGF-β superfamily and act as multi-functional growth mediators having the ability to stimulate cartilage/bone regeneration and formation process [77]. In a study involving rats, MSCs treated with PTE revealed that, during initial stages of cell proliferation, the *BMP4* mRNA and protein levels were high which confirms the proliferation effect of PTE via upregulation of BMP4 [25]. A higher level of p38 MAPK is associated with enhanced osteoblastogenesis [78]. The MAPK signaling pathway is responsible for altering different mediators like IGF1R, TRAF6, and STE20 which, in turn, affect the bone mineralization and regeneration process [79,80,81]. Recently it was reported that PTE stimulates osteoblasts’ differentiation and proliferation via the regulation of the MAPK signaling pathway in rat BMSCs [82]. The underlying pathway is the upregulation of IGF1R, STE20, and p38 MAPK expression and downregulation of TRAF6 expression [82]. Upregulation of p38 MAPK is also associated with an increased amount of immunomodulatory cytokines, such as IL-12 and IL-10 [83], which triggers Th2 and Th1 cell response that protects from infectious agents [84]. A recent study on bone MSC proliferation also revealed the upregulation of PI3K, AKT, and GSK3β and downregulation of β-CATENIN and TRAF2 protein expression after the administration of PTE [39]. The possible mechanism of the promotion effect of PTE on the stimulation of osteogenic differentiation is reliant on the Let7f-5p miRNA upregulation and TNFR2/PI3K/AKT signaling pathway [39]. Moreover, the TNFR2 is also considered as the main biomarker for curing cancer [85] osteoclastogenesis, inflammation, and many autoimmune disorders [86]. Enhanced levels of IL-6 and TNF-α in TD chicks depicted inflammation in the liver and other organs; however, PTE administration significantly reduced inflammatory cytokines. Fascinatingly, PTE suppressed the proinflammatory elements, suggesting that it can reduce inflammation and can be used for the treatment of bone mass loss and for the promotion of osteogenic differentiation [87].

## 5. Conclusions

Plastrum testudinis extract improved the antioxidant status and overall performance of the chickens exposed to thiram and acted as a bone healing and anti-inflammatory agent. Plastrum testudinis extract provided recovery to the TD-affected chickens by lowering the TD score and restoring the growth plate size and vascularization via the upregulation of the *AKT* and *PI3K* genes and protein expressions which reveals new target medicine for controlling and treating thiram-induced tibial dyschondroplasia.

## Figures and Tables

**Table 1:** Primers used for RT-qPCR.

Genes	Accession Number	Primer Sequence (5′-3′)	Product Size (bp)	Tm (°C)
*AKT*	AF039943	F: TGATGGCACATTCATTGGCTAC R: TGTTTGGTTTAGGTCGTTCTGTCT	122	58
*PI3K*	NM001004410	F: CGGATGTTGCCTTACGGTTGT R: GTTCTTGTCCTTGAGCCACTGAT	162	58
*GAPDH*	XM_019960295	F: CCTTCATTGACCTTCACTACATGGTCTA R: TGGAAGATGGTGATGGCCTTTCCATTG	127	58
